# Mir-29b in Breast Cancer: A Promising Target for Therapeutic Approaches

**DOI:** 10.3390/diagnostics12092139

**Published:** 2022-09-02

**Authors:** Silvia Grassilli, Valeria Bertagnolo, Federica Brugnoli

**Affiliations:** 1 Department of Translational Medicine, University of Ferrara, 44121 Ferrara, Italy; 2 LTTA Centre, University of Ferrara, 44121 Ferrara, Italy

**Keywords:** breast cancer, miR-29b, DNA methylation status, proliferation, invasion, angiogenesis, chemoresistance, radioresistance, targeted therapy

## Abstract

The miR-29 family comprises miR-29a, miR-29b, and miR-29c, and these molecules play crucial and partially overlapped functions in solid tumors, in which the different isoforms are variously de-regulated and mainly correlated with tumor suppression. miR-29b is the most expressed family member in cancer, in which it is involved in regulating gene expression at both transcriptional and post-transcriptional levels. This review focuses on the role of miR-29b in breast cancer, in which it plays a controversial role as tumor suppressor or onco-miRNA. Here we have highlighted the dual effect of miR-29b on breast tumor features, which depend on the prevailing function of this miRNA, on the mature miR-29b evaluated, and on the breast tumor characteristics. Remarkably, the analyzed miR-29b form emerged as a crucial element in the results obtained by various research groups, as the most abundant miR-29b-3p and the less expressed miR-29b1-5p seem to play distinct roles in breast tumors with different phenotypes. Of particular interest are the data showing that miR-29b1-5p counteracts cell proliferation and migration and reduces stemness in breast tumor cells with a triple negative phenotype. Even if further studies are required to define exactly the role of each miR-29b, our review highlights its possible implication in phenotype-specific management of breast tumors.

## 1. Introduction

Breast cancer (BC) is one of the most common malignancies; it affects mostly females and is a primary cause of cancer related mortality worldwide [1]. BC is a heterogeneous disease with multiple subtypes, based on clinical parameters and molecular profiling. Although each individual tumor is identified by the overall gene expression, the status of estrogen receptor (ER), progesterone receptor (PR), and human epidermal growth factor receptor 2 (HER2) in breast cancers define their treatment approach. In fact, whereas ER+ and HER2 + tumors can currently benefit from targeted therapies, triple-negative breast tumors (TNBC), lacking specific molecular targets, have chemotherapy as their only therapeutic option and constitute breast tumors with the worst prognosis [2].

In the last two decades, an increasing role in the occurrence and progression of BC has been assigned to non-coding RNAs, including micro-RNAs (miRNAs), long non-coding RNAs (lncRNAs), and circular RNAs (circRNAs), by virtue of their oncogenic and tumor-suppressive properties [3]. miRNAs are endogenous, small non-coding RNAs (19–23 nucleotides in length) which mainly work as post-transcriptional regulators of gene expression by binding to the 3′-untranslated region (3′-UTR) of target mRNAs and inducing their degradation or impairing their translation [4,5,6]. miRNAs play important roles in several biological processes, including organ development, cell proliferation, apoptosis, and differentiation [7,8,9,10]. As their targets include oncogenes and/or tumor suppressor genes, miRNAs can function as tumor promoters or suppressors, and, due to their tissue- and disease-specific expression, they are often ideal candidates as tumor biomarkers [11]. Furthermore, miRNAs may confer specific features to tumors, including resistance to radiation and chemotherapy, cancer stem cell (CSC) maintenance as well as augmented angiogenesis [12], therefore constituting prognostic markers and/or therapeutic targets [11,13].

Among the miRNAs de-regulated in multiple cancers, including breast cancer, the members of the miR-29 family are epigenetic modulators of DNA methylation that exert critical roles in carcinogenesis and cancer progression [14,15,16]. The miR-29b member acts as a crucial regulator in a variety of cancers, influencing epigenetic regulation, cell proliferation, apoptosis, differentiation, metastasis, and chemosensitivity [16].

This review aimed to ascertain the current known role of miR-29b in breast cancer, underlining its influence on key tumor characteristics, such as cell proliferation, metastatic potential, and response to chemo- and/or radiotherapy. Moreover, the controversial role of this miRNA in breast tumor cells and its potential as a molecular target in aggressive breast tumors will be discussed.

## 2. The miR-29 Family

The miR-29 family consists of miR-29a, miR-29b, and miR-29c, generated from the two primary transcripts, pri-miR-29a/b1 and pri-miR-29b2/c, derived from two gene clusters located on chromosomes 7q32.3 and 1q32.2, respectively. Mature miR-29a/b/c sequences show high similarity, but the different miRNAs have distinct subcellular localization [17,18] and exert individual functions [16,19,20,21]. Mature miR-29b includes miR-29b1 and miR-29b2, encoded by different genome regions but showing identical nucleotide sequence and function [22,23].

In addition to miR-29a-3p, miR-29b-3p, and miR-29c-3p, constituting the major forms that are most abundantly expressed, mature miR-29 also includes the minor forms miR-29a-5p, miR-29b1-5p, miR-29b2-5p, and miR-29c-5p [15]. Whereas the 3′ and 5′ arms are regulated by the same transcriptional mechanism, they differ in processing and maturation pathways, and often have opposite functions, even if only the most recent studies report complete details of the miR-29b members investigated [24,25,26].

The miR-29 family members are almost ubiquitous and have aberrant expression in various human cancers. Abnormal levels of miR-29b are common and have been correlated with clinicopathological characteristics in colorectal cancer [27,28], osteosarcoma [29], and breast cancer [30], suggesting its role in tumor features and its potential to function as a biomarker for diagnosis and prognosis, as well as a molecular target, in cancer.

Although most of the studies have been performed on leukemia cells, increasing evidence indicates the existence of both transcriptional and post-transcriptional mechanisms determining the various levels of the miR-29 family members in different solid tumors [31].

### 2.1. Transcriptional Regulation of miR-29b

Different binding sites for various transcriptional factors have been identified in the promoter regions of the miR-29a/b1 and miR-29b2/c clusters that are responsible for the peculiar ratio of miR-29a, miR-29b, and miR-29c expression in the different normal and tumor tissues.

Binding sites for the transcription factor c-Myc are present in the promoter region of both clusters [22,32] and chromatin immunoprecipitation (ChIP) experiments performed on breast cancer cell lines with different phenotypes clearly demonstrated that the binding of c-Myc to its promoter regions down-regulates miR-29b expression [33].

Binding sites for several transcriptional factors have been specifically identified in the promoter of miR-29a/b1 or miR-29b2/c clusters. In particular, the promoter region of the miR-29a/b1 cluster contains binding sites for Gli, CCAAT/enhancer-binding protein-α (CEBPα), T-cell factor/lymphoid enhancer factor (TCF/LEF), STAT1, NF-kB, and GATA3 [16,23]. Concerning NF-kB signaling, often activated in inflammation-related cancers, it may repress miR-29a/b1 promoter activity directly in several tumors, as in breast cancer [32,34], or indirectly, by activating the transcription suppressor Yin Yang 1 (YY1), as in rhabdomyosarcoma [35]. Also, GATA3 can induce miR-29b directly, through its binding with the miR29a/b1 promoter region, and, indirectly, by inhibiting the TGF-β and NF-kB pathways, in turn responsible of the down-regulation of miR-29b expression [32,35,36]. On the other hand, the reduced expression of miR-29b diminishes GATA3 levels, indicating the presence of a negative feedback between GATA3 and miR-29b [36].

In breast tumors, GATA3 is known to up-modulate miR-29b in ER+ tumor cells [36] but, recently, it has been shown that this transcription factor is not expressed in the triple-negative MDA-MB-231 cells, where miR-29b is transcribed by CEBPα [37], the main regulator of this miRNA in hematopoietic cells [23]. In addition, it has been found that CEBPα is expressed and regulates miR-29b only in triple-negative breast cancers belonging to specific molecular subtypes, and that its role is dependent on adequate levels of the multidomain protein Vav1, which promotes the association between CEBPα and the promoter region of miR-29a/b1 [37].

In breast cancer, different mechanisms affect the transcriptional regulation of miR-29b1-5p, the less expressed miR-29b1 form. In cancer cell lines with a basal-like, triple-negative phenotype, Milevskiy et al. demonstrated that BRCA1, known to bind promoters of numerous genes in human breast, affects the expression of miR-29b1-5p, consistent with the six binding sites for BRCA1 found inside the promoter region of miR-29a/b1 [25]. In addition, nuclear factor erythroid 2-related factor 2 (NRF2) was found to down-regulate miR-29b1-5p expression in MDA-MB-231 cells [26], according with data on acute myeloid leukemia cells, in which NRF2 binds antioxidant response elements (AREs) and inhibits miR-29b1 expression [38].

The promoter region of the miR-29b2/c cluster contains MyoD, transforming growth factor-β (TGF-β), Smad3, YY1, and PU.1 binding sites [39,40]. Furhermore, DNA damage can activate P53, which promotes the expression of miR-29 [41]. Remarkably, Smad3 binding to SBE elements interferes with the MyoD binding to the E-box sites that enables YY1 binding to the EZH2 and HDAC1 complex thus inducing a strong miR-29 repression [39]. YY1 binding sites associate with the Polycomb Group (PcG) transcriptional repressor factor EZH2 and with the histone deacetylase protein HDAC1 [35]. The loss of the binding of the repressive complex YY1/EZH2 during myogenesis determined the induction of miR-29. Moreover, by acting on the YY1/PcG complex, NF-kB induces epigenetic repression of miR-29b-2/c transcription in progenitor muscle cells [35]. NF-kB exerts an inhibitory effect on miR-29b2/c transcription also in breast-cancer-derived cells MCF7 and MDA-MB-231 by inducing the recruitment of YY1 onto the miR-29b promoter [34]. Two putative responsive elements of the myeloid master regulator PU.1 (SPI1) were identified in the promoter of the miR-29b2/c cluster and low expression of miR-29b in APL cells is due to aberrant expression of this transcription factor [42]. Recently, it was demonstrated that the recruitment of PU.1 to both miR-29b promoters in APL-derived cells is dependent on the levels of Vav1 [43], highlighting the role of this protein in regulating miR-29b in different cell tumor models.

Recently, FOXO3a has been reported to directly up-regulate miR-29b2 in MDA-MB-231 cells, according with the presence of two FOXO3a-binding sites (FHRE-1, FHRE-2) on the promoter region of miR-29b2/c [44].

**Figure 1:**
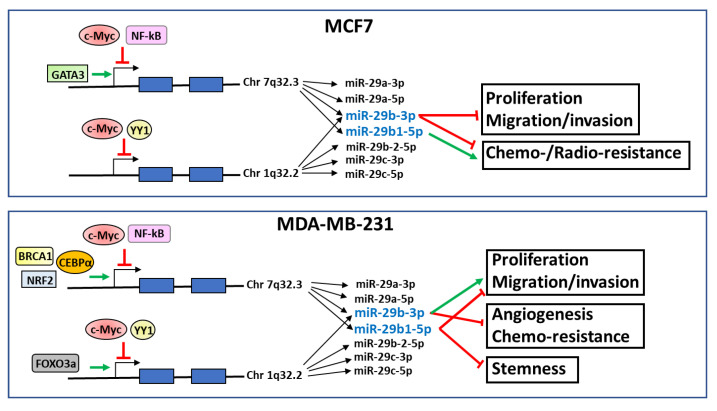
Schematic representation of transcription factors that specifically promote (green arrows) or inhibit (red blocked lines) miR-29a/b1 or miR-29b2/c expression and known effects of miR-29b-3p and miR-29b1-5p on the reported cell functions in MCF7 and MDA-MB-231 breast cancer cell lines.

The transcription factors known to interact with the promoter regions of miR-29a/b1 and miR-29b2/c clusters in MCF7 (ER-positive) and MDA-MB-231 (triple-negative) breast tumor cells are summarized in Figure 1.

### 2.2. Post-Transcriptional Regulation of miR-29b

The post-transcriptional regulation of the members of the miR-29 family involves long non-coding RNAs (lncRNAs), RNAs that exert their roles mainly as competitive endogenous RNAs via sponging miRNAs, thereby reducing their availability to target mRNAs [45]. Several lncRNAs have been reported to contribute to tumorigenesis and cancer progression, and lncRNA–microRNA–mRNA signaling axes have recently been shown to play a key role in the development of tumors [45,46]. To date, different lncRNAs are known to regulate miR-29 expression. LncRNA H19 regulates epithelial-to-mesenchymal transition (EMT) and metastasis of bladder cancer as competing endogenous RNA of miR-29b-3p [47]. In cervical cancer cells, the lncRNA HOTAIR binds with miR-29b, down-modulating its level and resulting in enhanced EMT, migration, proliferation, and chemoresistance [48]. LncRNA PVT1 promotes the malignant progression of AML and is correlated with poor prognosis, via sponging the miR-29b family [49].

No data are available concerning specific lncRNA involved in post-transcriptional regulation of miR-29b in breast tumors. On the other hand, recently, circular RNAs, extremely stable forms of non-coding RNAs, have been discovered to function as miRNA sponges and, consequentially, modulators of gene expression in breast tumor cells [50]. In invasive breast cancer cells with different phenotypes, miR-29a-3p and miR-29b1-3p are sponged by circACAP2, promoting the expression of COL5A1, one of miR-29a/b-3p targets [50].

Among the mechanisms responsible for the levels of miR-29b members in the different cell types, their degradation process also plays an important but not yet fully understood role. The decay dynamic varies considerably between individual miRNAs and the position of specific nucleotides seems critical for this process. For instance, uracils at nucleotide positions 9–11 of miR-29b play a crucial role in its rapid turnover in cycling HeLa cells [51].

## 3. Roles of miR-29b in Breast Cancer

miR-29b expression has been detected in all groups of malignant and benign breast carcinoma, and this miRNA is down-regulated in ductal with respect to lobular tumors [30]. In invasive breast tumors, miR-29b levels show a relationship with ER status, being up-regulated in ER-positive breast tumors compared with their basal-like and HER2-enriched counterparts [30]. Interestingly, high miR-29b seems to promote and preserve the luminal differentiation of tumor cells through down-regulation of a network of pro-metastatic regulators [52].

miR-29b was also correlated with tumor stage, and, apart from the work of Wang et. al., which showed that miR-29b expression is often increased in breast cancer patients with armpit lymph metastasis [53], low miR-29b levels were positively associated with larger tumor sizes and more advanced stage [30,54]. Notably, low expression of miR-29b in ductal carcinoma has been associated with poor prognosis, in terms of overall survival (OS) and disease-free survival (DFS) in the ER-positive tumors, and OS in ER-negative cases [55]. The expression levels of miR-29b were also investigated in breast tumours arising with BRCA1 mutation that do not commonly express ER, PR, or HER2 and have decreased chances of survival and no targeted therapies available. The analysis of breast tumors from the METABRIC and TCGA cohorts demonstrated that the less expressed miR-29b-1-5p greatly stratified survival with high expression associated with a better outcome for patients with basal-like tumours and TNBC [25], suggesting that this miRNA is a powerful biomarker for predicting patient outcomes in all the subtypes of breast cancer. A more recent study on patients of “KM plotter database” confirmed a better prognosis for patients affected by invasive breast carcinoma expressing elevated levels of miR-29b-3p [56], specifically correlating breast tumor prognosis with the more abundant form of miR-29b.

Although most of the literature indicates a positive correlation between miR-29b levels and the malignancy of breast cancers, summarized in Table 1, identifying the mechanism/s by which this miRNA acts in breast cancer cells appears particularly complex. In fact, the functions of miR-29 family members, especially of miR-29b, are various and seem to depend on tissue localization and on both transcriptional and post-transcriptional regulatory processes [16]. In addition, as reported in other solid tumors [57], the role of the more stable miR-29b-3p and of the less expressed miR-29b1-5p in breast tumor cells seems to be different, and sometimes opposite [24,26].

Lastly, miR-29b is elevated in the ER+ (luminal molecular subtypes) compared with TNBC (basal-like) and HER2-enriched breast tumors [30], indicating that different mechanisms must be activated in breast cancer cells with different phenotypes. Accordingly, the large majority of the studies on the role of miR-29b in breast cancer have been conducted on the well-known luminal MCF7 and triple-negative MDA-MB-231 cell lines.

### 3.1. MiR-29b as a Controller of DNA-Methylation Status

Aberrant DNA methylation and changes in gene expression secondary to methylation-dependent gene silencing occur frequently and contribute to initiation, development, and progression of cancers, including breast tumors [58,59,60,61]. Several miRNAs have been identified as both regulators of DNA methyltransferase (DNMT) levels and targets of aberrant DNA methylation in breast tumors [62]. miR-29b was classified as an epi-miRNA, a term proposed to indicate miRNAs with the ability to repress enzymes involved in epigenetic machinery [63]. miR-29b may directly or indirectly target DNMTs and/or regulate members of the DNA demethylation pathway, leading to the downregulation of global DNA methylation status [64]. The ten-eleven translocation (TET) family of DNA demethylases [65] protects against aberrant demethylation [66]. Interestingly, whereas miR-29b directly targets DNMTs, it may also repress the activity of TET1, which has opposed roles in controlling the status of DNA methylation [66]. This suggested that miR-29b may primarily function as a stabilizer of DNA methylation status, thereby suppressing tumorigenesis. Moreover, to maintain the balance between DNA methylation and demethylation, miR-29 members may produce demethylation effects, acting as oncogenes [67].

An example of how the indirect activity of miR-29b on methylation status may promote tumor features was revealed in MDA-MB-231 and MCF7 cells, in which it has been demonstrated that miR-29b can directly target the 3′UTR region of TET1, in turn responsible for direct binding with the promoter region of the EMT-related transcription factor ZEB2 [68]. Due to the demethylation activity of TET1, its knockdown results in the up-regulation of ZEB2 expression as well as in up-modulation of the methylation level of the ZEB2 promoter, allowing promotion of malignant progression in tumor cells [68]. In the MDA-MB-231 cell line, the study of De Blasio and colleagues reported that the over-expression of miR-29b1-5p caused a decrease in DNMT1, DNMT3A, and DNMT3B levels, and the changes in the methylation status of their promoters allow the expression of tumor suppressor genes as HIN1, RASSF1A, and CCND2 [26].

### 3.2. MiR-29b Regulates Cell Proliferation, Motility, and Metastasis

As reported by Kwon and colleagues, miR-29 family members act as tumor suppressors in more than 95% of publications on various human hematologic malignancies and solid tumors [69]. In strong contrast to the role as tumor suppressors, miR-29b was also described to function as an oncogene, via the negative inhibition of tumor suppressor genes or by interfering in pathways that control cellular responses [69]. For this reason, the overall role of miR-29b in breast cancer cells is not completely understood and its relationship with breast carcinogenesis remains controversial.

#### 3.2.1. miR-29b as a Tumor Suppressor miRNA

Accumulating evidence demonstrated that the expression level of miR-29b is deregulated in almost all primary breast tumor tissues and cell lines and that its re-expression induces a reduction of malignancy in terms of cell proliferation, migration, and invasion [21,30,55]. In the MCF7 cell model, it was reported that the over-expression of miR-29b decreases mRNA levels of SPARC [70], also known as osteonectin or BM-40, an albumin-binding glycoprotein secreted by cells and modulating their interactions with extracellular matrix (ECM) to down-regulate cell proliferation, migration [71,72], and metastasis [14]. In addition, tumor microarray analysis and luciferase assays allowed identification of C1QTNF6, SPARC, and COL4A2 as direct targets of miR-29b in MCF7 cells and involved in their invasion. Furthermore, the up-regulation of miR-29b reduces cell proliferation and induces the apoptosis of MCF7 cells by regulating the expression of STAT3 [54]. It has been recently demonstrated that, in certain molecular subtypes of triple-negative breast breast tumor cells, miR-29b-3p down-modulates Akt2, a member of the Akt family mainly involved in migration and metastasis of breast cancer, and prevents the in vivo lung colonization of MDA-MB-231 cells [73].

As reported above, circACAP2 may promote in vitro cell proliferation and migratory and invasive abilities of breast tumor cells with different phenotypes by sponging both miR-29a-3p and miR-29b-3p, thus preventing the silencing effect of both miRNAs on their target COL5A1 gene, which encodes the α1 chain of type V collagen [50].

A number of recent studies specifically assigned to miR-29b1-5p the antitumor role played by miR-29b in breast-tumor-derived cells with a triple-negative phenotype. Drago-Ferrante and colleagues demonstrated that miR-29b1-5p expression is strongly down-regulated in TNBC tissues and cell lines and this impacts on proliferation and migration of TNBC-derived cells [24]. They found that the over-expression of miR-29b1-5p in MDA-MB-231 cells decreases in vitro growth rate, Ki-67 levels, and migration [24], and the decrease of Wnt/βcatenin and Akt-signaling pathways, both involved in tumorigenesis and progression of breast cancer [74,75]. In addition, it was demonstrated that miR-29b1-5p down-modulates, at both mRNA and protein levels, spindlin 1 (SPIN1), which enables methylated histone binding activity and participates in PI3K/Akt-mediated chemoresistance of metastatic breast cancer [24,76]. The study of De Blasio and colleagues reported the existence of a regulatory loop including miR-29b1-5p and NRF2, able to regulate the fate of the MDA-MB-231 cell line [26]. In particular, the over-expression of miR-29b1-5p seems to exert opposite effects with respect to NRF2 by increasing the generation of reactive oxygen species (ROS) and inducing a decrease in p-Akt levels, in turn responsible for reducing cell proliferation [26]. Furthermore, miR-29b1-5p over-expression in the same cell model decreased the mRNA and protein levels of key stemness genes, such as OCT4, SOX2 and NANOG, implicated in the regulation of self-renewal of CSCs [24]. Interestingly, in tertiary mammospheres from MDA-MB-231 cells, the expression of miR-29b1-5p dramatically decreases, suggesting its inverse correlation with stemness [24].

#### 3.2.2. MiR-29b as an Onco-miRNA

Wang and colleagues reported that miR-29b expression is elevated in highly metastatic breast cancer cells and tissues in comparison with lowly metastatic breast cancer. They also revealed that miR-29b expression is often higher in primary tumors of breast cancer patients with lymph node metastasis [53]. A functional role of miR-29b in promoting cell migration and invasion in breast tumor cell lines with different phenotypes was proposed, and the downregulation of PTEN was identified as the mechanism contributing to tumor metastasis [53]. Furthermore, greater expression of miR-29b-3p was reported in BC with low DNA repair capability [76,77].

More recently, Zhang and colleagues reported that miR-29b-3p is highly expressed in triple-negative MDA-MB-231 cells, compared with the ER-positive MCF7 and the non-transformed MCF-10A cell lines, and that its inhibition suppresses in vitro cell proliferation, migration, and invasion, and induces apoptosis [78]. In MDA-MB-231 cells, this was correlated with the direct target of TRAF3, reported to inhibit the activation of the NF-κB signaling pathway [78]. In addition, and as mentioned above, miR-29b may have an oncogenic role due to the loss of its regulatory activity on genes involved in the modification of DNA methylation status [67], allowing up-regulation of EMT-related genes [68].

### 3.3. MiR-29b Reduces Tumor Angiogenesis

miRNAs are key players in the epigenetic orchestration of angiogenesis, a process by which new blood vessels are formed to supply oxygen and nutrients inside tumors. The angiogenic process is regulated by a balance between pro- and anti-angiogenic stimuli involved in the degradation of the vascular basement membrane and remodeling of ECM, in endothelial cell migration and proliferation, and in generation of new matrix components [12]. Moreover, the angiogenic switch and the tumor neovascularization may be due to hypoxic conditions or the consequence of therapies promoting tumor progression [12]. Various studies have demonstrated the key participation of MMPs along with EMT to promote angiogenesis, infiltration by cancer cells, and metastasis [79]. MMPs participate in the degradation of ECM components, including the basement membrane and the tumor surface, resulting in tumor cell migration into the near tissue. Furthermore, MMPs promote tumor growth and spread through the capillary endothelium and neovascularization [79].

As already demonstrated in other solid tumors, miR-29b could affect the expression of several members of MMPs family in breast cancer cell lines. In particular, the over-expression of miR-29b suppresses tumor angiogenesis and metastasis by directly regulating the expression of MMP2 [80]. Moreover, in MDA-MB-231 cells, miR-29b significantly down-regulates a set of pro-metastatic genes involved in angiogenesis, collagen remodeling and matrix degradation, including MMP2, MMP9, PDGF, and VEGF [36]. The latter is a growth factor, over-expressed in various cancers, that binds to and activates both VEGFR-1 and VEGFR-2, promoting vasculogenesis and angiogenesis in breast cancer [81]. In MDA-MB-231 cells, in vivo miR-29b treatment inhibits VEGF expression without inducing HIF1-α, a master regulator of VEGF, indicating that the over-expression of miR-29b may interfere with the canonical regulation of VEGF [81]. In the same cell model, miR-29b can reduce the expression of VEGF and also inhibits tumor vascularization by targeting Akt3, that in turn regulates the expression of VEGF and C-Myc [82]. Still, in MDA-MB-231 cells, miR-29b-2 targets VEGF-A and cooperates with miR-338 to inhibit invasion and metastasis in both in vitro and in vivo models [44]. Furthermore, the reduction in miR-29b/miR-338 expression induced by inactivation of the transcription factor FOXO3a inhibits the targeting of VEGF-A/NRP1, contributing to the aggressive behavior of breast cancer [44].

### 3.4. MiR-29b Regulates Chemo- and Radiosensitivity in Breast Cancer

The resistance of cancer cells to conventional chemotherapeutics and radiotherapy represents the major obstacle for cancer treatment and the main reason for therapy failure [83]. In breast cancer, especially in triple-negative tumors, chemotherapy is the most effective and often the only therapeutic strategy, but the presence of drug resistance, which may lead to tumor recurrence and metastasis, and is associated with poor prognosis, reveals that molecular mechanisms at the basis of chemosensitivity are mostly unknown [84].

As in other solid tumors, several studies reported a regulatory role for different miRNAs [84,85], and some miRNA expression profiles are associated with resistance to chemotherapy in breast tumors [86,87]. Zhou et al. revealed that MCF7 breast cancer cells resistant to Adriamycin have significant over-expression of miR-29b-3p and miR-29b1-5p [88]. Mimics or inhibitors of miR-29b1-5p could modulate the drug resistance of MCF7 cells to Adriamycin regulating the IC50 of the drug, and treatment with liposomal curcumin, down-regulating the expression of miR-29b1-5p, may reverse the resistance of MCF-7 cells [88]. At variance, Ji and colleagues demonstrate that miR-29b-3p promotes sensitivity of luminal or HER2-positive breast cancer cells to Palbociclib [33], a selective inhibitor of CDK4 and CDK6 kinases, which could prevent progression of the cell cycle from G1 into the S phase in various tumors [89]. In vitro studies demonstrated that MDA-MB-231 cells transfected with miR-29b-3p mimics are more sensitive to Palbociclib and that loss of miR-29b-3p in MCF7 and SK-BR-3 cells induced the resistance to Palbociclib treatment [33]. ChIP analysis revealed that the transcription factor c-Myc may bind the miR-29b-3p promoter, allowing identification of a c-myc/miR-29b-3p/CDK6 axis that might be responsible for Palbociclib insensitivity, in which c-Myc activation resulted in down-regulation of miR-29b-3p, which enhanced CDK6 expression [33]. In the same cell models, miR-29b-3p could negatively modulate CDK6 expression by directly targeting its 3′-UTR, inducing cell cycle arrest at the G1 phase [87].

Concerning radiosensitivity, it has been well established that cellular exposure to radiation results in damage of DNA and other cellular structures, inducing a complex cascade of downstream pathways in both the cytoplasm and nucleus, involved in modulation of cell cycle, DNA repair, ROS defense, cytokine production, and apoptosis [90]. Individual responses to radiotherapy (RT) vary among disease types and patients [91] and the resistance to RT is associated with several biological alterations of the tumor cells and with the tumor microenvironment [92].

In solid tumors, increasing evidence shows that several miRNAs, including miR-29b, play a crucial role in the cellular response to ionizing radiation and in the regulation of radioresistance mechanisms [93,94]. Despite the role of miR-29b in regulating radiosensitivity in different tumors [95,96,97], very little is known in mammary tumors. A recent work by Pan and colleagues demonstrated that over-expression of miR-29b-3p significantly enhanced radiosensitivity in 3D-cultured MCF7 cells and in in vivo models, whilst the knockdown of miR-29b-3p enhances radioresistance [56]. It was proposed that miR-29b-3p regulates radiosensitivity by inhibiting the kinetic process of DNA damage repair followed radiation, by decreasing the expression of DNMT3B, Bcl-2, PI3KR1, Akt2, and RBL1 [56].

## 4. Circulating miR-29b

It is well recognized that extracellular vesicles (EVs), including exosomes, may contain proteins, RNA, and DNA and are secreted from viable cells into the blood circulation [98]. Recently, also miRNAs can be stably detected in the blood, as they are resistant to RNAase digestion and to many severe environmental conditions [99], and circulating miRNAs are often differentially expressed in the serum/plasma of patients with different pathologies, including cancers [100,101]. As the study of miRNAs in blood is a simple, affordable, minimally invasive, and time-saving detection method for the early diagnostic and prognostic evaluation, exosome-encapsulated miRNAs might represent ideal biomarkers for early diagnosis, for cancer screening, or for evaluating disease progression and treatment efficacy [102,103].

Concerning miR-29b, it was detected in human serum and differentially expressed in the blood of patients with neurodegenerative diseases, including Alzheimer’s disease (AD), in which it constitutes a potential biomarker for AD treatment [104]. Furthermore, circulating miR-29b was reported to increase with aging, which often constitutes a comorbidity in breast cancer, and seems to be correlated with early atherosclerosis [105], muscle atrophy [106], changes in cortical metabolism [107], and osteoporosis [108].

Circulating miR-29b is often deregulated in the sera of tumor patients, and its diagnostic and/or prognostic potential shows great variability between different tumors. Serum levels of miR-29b were down-regulated in patients with neuroendocrine tumors (NET), in which it constitutes a diagnostic but not prognostic biomarker [109]. Instead, miR-29b-3p has been found to be significantly overexpressed in EVs from patients with prostate cancer (PCa) [110], in which it constitutes an early diagnostic marker [111]. Increased serum levels of miR-29b have been correlated to poor overall prognosis in neck cancers (HNC) [112]. At variance, high circulating levels of miR-29b-3p were positively correlated with DFS and OS of metastatic colorectal cancer patients treated with Bevacizumab [113]. Likewise, serum concentrations of miR-29b in cholangiocarcinoma (CCA) were significantly elevated compared with healthy controls, but do not reflect disease characteristics and patient prognosis, and a postoperative decrease was associated with a good prognosis. [114]. Finally, the serum levels of miR-29b increase in osteosarcoma and were correlated with clinicopathological characteristics and patient prognosis [29].

Concerning breast cancer, miR-29b2 was upregulated in the sera of breast cancer patients, without differences between the type of tumors but with a positive correlation with cancer progression, suggestive of its use as a diagnostic and prognostic marker [101]. Furthermore, miR-29b is part of a panel of exosomal miRNAs that may constitute valuable biomarkers for predicting breast cancer recurrence [102]. More recently, Li et collaborators revealed that miR-23a-3p, but not miR-29b2-5p, is downregulated in the plasma of patients with BC compared with healthy control individuals and these miRNAs are significantly associated with sex hormone receptor, clinical stage, and lymph node metastasis [115].

As in tissues [116], circulating levels of miR-29b have been reported to be modified by diet or by specific microelements, including polyphenols, according with the relationship of miR-29 with NRF2 [26], a redox sensitive transcription factor induced by dietary phytochemicals and regulating the transcription of a number of proteins with antioxidant and anti-inflammatory functions [117]. Although nothing is known in breast cancer, the consumption of green tea polyphenols (GTP)s was recently reported to increase the levels of miR-29b in blood, which seems to be correlated with a reduced susceptibility for developing lung cancer [118]. Moreover, it has also been demonstrated that regular consumption of GTPs reduces ultraviolet B (UVB)-radiation-induced murine non-melanoma skin cancers (NMSCs), and blocks UVB-induced miR-29b depletion [119], suggesting that diet may have an important role in cancer prevention.

## 5. Conclusions

This is the first review that collects data concerning the role of miR-29b in breast cancer, in which, despite accumulating evidence correlating high levels of this miR-29 member with the reduced malignancy of breast tumors, it seems to play a controversial role in breast tumor cells. We conceive that some discordance may be at least in part attributed to different sample sizes, to heterogeneity between the same cell line [120] or to the use of techniques with different discriminant capability. On the other hand, the actual dual role of miR-29b as a suppressor or promoter of breast tumor cell malignancy can be ascribed to its >involvement in the epigenetic regulation of DNA methylation status. From imbalance between down-regulation of DNMTs and of the DNA demethylases TET1, having opposite roles in controlling DNA methylation, it may result that miR-29b acts as a tumor suppressor or as an oncogene, depending on cell conditions.

Interestingly, from the analysis of the studies produced in the last few years, the evaluated form of miR-29b emerged as a crucial element in the results obtained by the various research groups. In fact, specific evaluation of individual miR-29bs revealed that miR-29b-3p and miR-29b1-5p play different roles in breast tumors with different phenotypes (Figure 1). In particular, miR-29b1-5p seems to play an antitumor role in breast tumor cells with a triple-negative phenotype, in which this miRNA counteracts proliferation, migration, and the maintenance of stem cell characteristics.

Even if further studies will be required to better characterize the expression and the role of each miR-29b form in the different breast tumor subtypes, our review highlights its possible implication also in the therapeutic approach to breast tumor, to specifically counteract phenotype-related malignant features and/or chemoresistance.

## Figures and Tables

**Table 1:** Positive (pos) or negative (neg) correlation of miR-29b in invasive breast tumor tissues with tumor malignancy.

Authors	Patients n°	Tumor Size	Lymph Methastasis	OS	DFS
Wang et al., 2011 [53]	20	-	pos	-	-
Shinden et al., 2015 [55]	94	neg	-	pos	pos
Qin et al., 2015 [54]	67	neg	none	-	-
Milevskiy, et al., 2018 [25]	METABRIC	-	-	pos	-
Milevskiy, et al., 2018 [25]	TCGA	-	-	pos	-
Papachristopoulou et al., 2018 [30]	121	none	-	-	pos
Pan et al., 2021 [56]	1262	-	-	pos	-

## Data Availability

Not applicable.
